# Roles of Proteins/Enzymes from Animal Sources in Food Quality and Function

**DOI:** 10.3390/foods10091988

**Published:** 2021-08-25

**Authors:** Chenyan Lv, Chen Xu, Jing Gan, Zhenghui Jiang, Yumeng Wang, Xueli Cao

**Affiliations:** 1 Beijing Advanced Innovation Center for Food Nutrition and Human Health, Beijing Technology and Business University (BTBU), Beijing 100037, China; 2019023@cau.edu.cn (C.L.); xuchen5140@163.com (C.X.); goodbai0427@163.com (Y.W.); 2 College of Life Sciences, Yantai University, Yantai 264005, China; keaiganjing@126.com; 3 Beijing Key Laboratory of Functional Food from Plant Resources, College of Food Science and Nutritional Engineering, China Agricultural University, Beijing 100083, China; zhenghui0124@126.com

**Keywords:** protein, animal food, food quality, interactions

## Abstract

Animal proteins are good sources of protein for human, due to the composition of necessary amino acids. The quality of food depends significantly on the properties of protein inside, especially the gelation, transportation, and antimicrobial properties. Interestingly, various kinds of molecules co-exist with proteins in foodstuff, and the interactions between these can significantly affect the food quality. In food processing, these interactions have been used to improve the texture, color, taste, and shelf-life of animal food by affecting the gelation, antioxidation, and antimicrobial properties of proteins. Meanwhile, the binding properties of proteins contributed to the nutritional properties of food. In this review, proteins in meat, milk, eggs, and fishery products have been summarized, and polysaccharides, polyphenols, and other functional molecules have been applied during food processing to improve the nutritional and sensory quality of food. Specific interactions between functional molecules and proteins based on the crystal structures will be highlighted with an aim to improve the food quality in the future.

## 1. Introduction

Animal foods are good source of “high quality” protein for humans, especially lean meat, milk, eggs, and fishery products [1]. Diet composition improvement represents a key factor to enhance the health status and welfare of animals [2,3] as well as to enhance productivity and performance in livestock [4,5]. Proteins have been widely used as food components in the food industry due to their nutritional value and functional properties [6]. In the past several years, chemical modifications such as oxidation and glycosylation have been developed to improve the functional properties [7]. It is worth mentioning that gelation, oxidation, and assembly of proteins occurred which may affect the texture, nutrition, and sensory properties of food during food processing and storage [8]. Recent research has mainly focused on the improvement of gelation, inhibition of oxidation and assembly of proteins. Taking advantage of the antioxidant properties of polyphenols, the protein-polyphenol interactions have been reported to help inhibit or slow down the oxidation of protein from meat [9], and further affect the gelation process.

The micro- and macroscopic structures of food determine the behaviors of food. Proteins, especially myofibrillar protein in meat, contribute most to the formation of three-dimensional network structure in processed meat products, which will help to hold water inside and confer meat with a “juicy” taste. On the other hand, protein gelation also contributes to the quality of dairy products [10]. More interestingly, the gelation process can be improved by other molecules such as polysaccharides and polyphenols. Meanwhile, proteins in milk such as bovine serum albumin and lactoferrin play crucial roles in nutrient transportation [11]. The interactions between proteins and substances from environmental pollution or molecules produced during food processing may affect the transporting efficiencies to some extent [12].

In addition to the interactions between protein and small molecules, the antibacterial activities and enzymatic activities of proteins or enzymes also affect the product quality especially the storage stability and shelf-life. Therefore, it is imperative to summarize the roles of proteins from animal sources in food quality, and the effects of interactions between proteins and small molecules on food texture, and sensory and nutritional properties. As for the selection of research papers, we have analyzed the main proteins/enzymes affecting food qualities in each kind of food, which are myofibrillar protein, hemoglobin, whey protein, bovine serum albumin, casein, lactoferrin, lysozyme, ovalbumin, collagen, polyphenol oxidase, and ferritin (Table 1). Thus, we focused on these proteins and the interactions affecting quality. This review will not only provide guidelines for food processing to improve the quality of food products, but also make it possible to increase stability by regulating the properties of proteins or enzymes.

## 2. Proteins in Meat

### 2.1. Myofibrillar Protein

**Figure 1:**
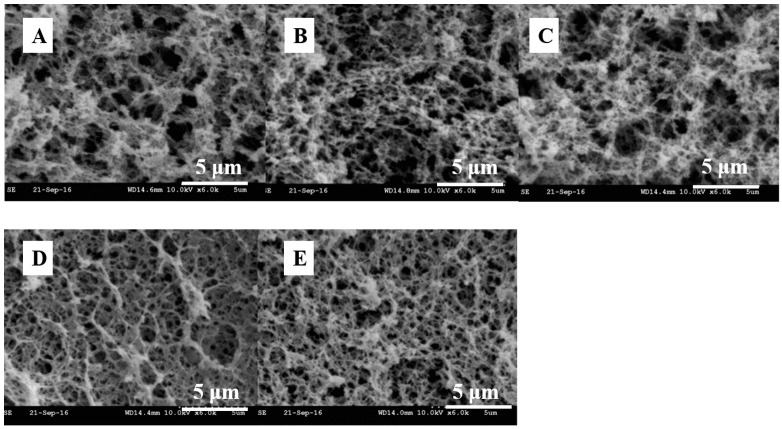
SEM microstructure of MPs gels induced by different combinations of pressure and heating; scale bars are 5 μm. (**A**). 0.1 MPa, 75 °C, 30 min; (**B**). 200 MPa, 20 °C, 30 min, then 0.1 MPa, 75 °C, 30 min; (**C**). 400 MPa, 20 °C, 30 min, then 0.1 MPa, 75 °C, 30 min; (**D**). 200 MPa, 75 °C, 30 min; (**E**). 400 MPa, 75 °C, 30 min. This figure was cited from reference [44].

Myofibrillar protein (MP) is the main protein in meat, accounting for 55–60% of the total [42], and predominately consisting of myosin, actin and some other regulatory proteins. Numerous myofibrils formed the muscle fibers and further joined together by connective tissue to form muscle. Most actin exists in the muscles as monomer polymerized double-stranded helical strands and can combine with the head of myosin to form thick filament. Myosin, which consists of two identical heavy chains, is an end-polarized double-headed long-chain molecule with helical structure. Assembly of myosin to filaments makes it hardly soluble, and limits its applications in the food industry. At present, strategies to inhibit or disrupt myosin formation have been widely developed [43]. In meat processing, heat-induced gelation of MP in muscle is a very important feature [8,44], the first step of which is to denature and unfold the protein, so that the reaction group is exposed, especially the hydrophobic group of myosin. Then, the unfolded proteins are gathered, and the attraction and repulsion between adjacent polypeptide chains are balanced. After that, a stable three-dimensional network gel structure was finally formed [7]. As shown in Figure 1, the microstructure of gels heated under different pressures was observed by scanning electron microscopy, and thinner fibers were observed under 200 MPa than under 400 MPa [44]. Generally, the forces that maintain the protein structure and its gel network mainly include electrostatic interaction, hydrophobic interaction, hydrogen bond, disulfide bond, etc. As the main protein of MP, myosin is essential to form filamentous gel structure. Other MPs such as actin do not form gels, but have an important impact on the viscoelasticity of gels [13].

**Figure 2:**
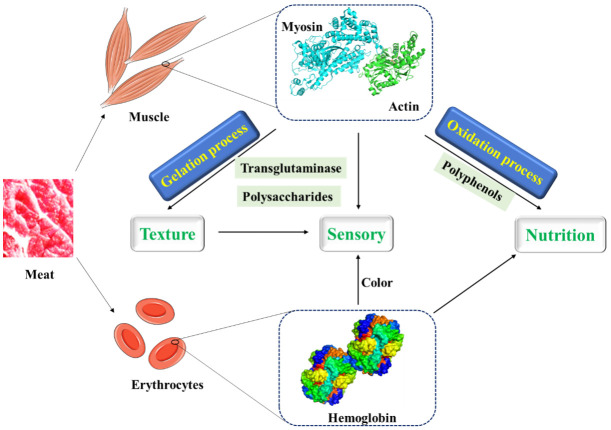
The interactions between proteins in meat and other molecules and their food texture, sensory, and nutritional effects.

At present, a variety of substances are used to improve the structure of MP gel to produce meat products with better quality and taste (Figure 2). Transglutaminase is a substance that promotes the widespread use of meat protein gels. A non-disulfide covalent bond is formed by transglutaminase between glutamine residues and lysine residues within and between proteins to cross-link protein molecules, thereby improving the properties of protein gels [45,46]. Moreover, some plant proteins which can cross-link with MP, form more stable gels by hydrophobic interactions and disulfide bonds, and improve the cooking yield of MP gels under the induction of glutamic amide transaminase, have been applied to improve the texture and sensory properties of products in meat processing [47,48,49,50,51,52].

In addition to macromolecules, polyphenols as small molecules also interact with MP in meat processing. Polyphenols not only have antioxidant and flavoring effects on MP, but also have important effects on gelation and emulsification [53]. Guo et al. [54] found that gallic acid can promote myosin cross-linking to form a denser and more uniform gel structure catalyzing by glucose oxidase. Moreover, the addition of catechins has been identified to increase the hydrophobicity of myofibrillar protein surface while reducing the thiol content, and Jia et al. [55] also confirmed the adverse effects of catechins on myofibrillar protein gel properties. By studying the effects of five phenolic compounds in mulberry polyphenols on the structure and functional properties of myofibrillar protein, Cheng et al. [56] found that all the five would affect the quality of meat products, in which caffeic acid and rutin played a key role. However, some polyphenols may play double functions during MP gelation [9,57]. A certain concentration of polyphenols (6 and 30 μmol/g protein of gallic acid and chlorogenic acid) can promote cross-linking between proteins and improve the performance of MP gel, but high concentrations of polyphenols (150 μmol/g protein of gallic acid and chlorogenic acid) will lead to excessive covalent binding and excessive protein aggregation, which further inhibits the formation of gel.

### 2.2. Hemoglobin

As a secondary product of meat products, blood contains a substantial amount of high-quality proteins that are not used [58,59]. Hemoglobin as the main component of protein in blood is composed of globin and porphyrin iron, and is rich in iron ions and amino acids. It can be used as a natural iron supplement and has the characteristics of easy absorption [19]. Hemoglobin has good solubility, emulsifying, foaming, and swelling properties, but its application in food is limited due to poor stability, dark color and metallic flavor [18]. By means of Maillard reaction, acetylation, deamidation, and succinylation, the structure of protein can be changed to improve its properties and function. Maillard reaction, which does not require additional chemicals, is considered to be the best method to improve the functional properties of food proteins [6]. Unlike Maillard reaction, adding glucose as reducing agent, niacin or nicotinamide as a chelating agent to porcine hemoglobin for spray drying can effectively prevent hemoglobin from self-oxidation and stabilize color [60]. Meanwhile, nitrosohemoglobin as a colorant can partially replace nitrite to improve the safety of meat products. After glycosylation, the stability of nitrosohemoglobin will be improved [61,62]. Liquid or dry blood treated with carbon monoxide can also be used as colorants in meat products, which has been proved to be feasible in mortadella [63,64]. More importantly, tea polyphenols can also affect the stability and color of hemoglobin, which is connected with the concentration of tea polyphenols [65]. At low concentrations, tea polyphenols can inhibit the oxidation of heme iron and maintain the hemoglobin color. High concentrations of tea polyphenols may lead to hemoglobin structural damage and heme iron exposure, thereby promoting hemoglobin oxidation discoloration.

In order to improve the utilization rate of hemoglobin, researchers decolored the hemoglobin and separated the globin by acidified acetone, carboxymethyl cellulose, sodium alginate and enzymatic hydrolysis [66,67,68,69]. Silva [70] compared the effects of globin extracted by acidified acetone (AG) and carboxymethyl cellulose (CG) and sodium caseinate (CA) as emulsifier agent on the quality characteristics of raw and cooked ham pâté. It was found that CG increased the salt-soluble protein content and the stability, CA increased the salt-soluble protein content and water holding capacity, and AG was unfavorable for these three parameters. More interestingly, Shi et al. found that as a histidine-specific protease, Aspergillus glutamic acid peptidase (AGP) could effectively decolor hemoglobin, and the AGP-decolored hemoglobin hydrolysates (AGP-Hb) had good emulsification, foaming, and water binding.

### 2.3. Interactions between Meat Protein and Other Factors Affecting the Quality of Meat Product

Meat is a very efficient deliverer of protein. In addition to fresh meat, meat is usually further processed to improve the sensory quality and to extend their shelf life. During food processing, large amounts of polysaccharides such as κ-carrageenan and flaxseed gum have been used in comminuted meat products to improve their functional properties such as emulsification, fat, and water binding capacity, while improving their texture and appearance.

In low-fat meat products, polysaccharides acting as water binders affect the thermal transition temperatures of meat proteins by interacting with meat protein. The electrostatic force in nature is the principal force responsible for these interactions. Meanwhile, by studying the thermal properties, dynamic rheological properties, texture and microstructure of salt-soluble meat protein and flaxseed gum, it was found that the interaction between salt-soluble meat protein and flaxseed gum occurred, and the main force for the formation and stabilization of protein-polysaccharide gels seemed to be electrostatic force [71,72]. The gel formed by flaxseed gum is thermoreversible, which is significantly related to the flavor release, perception of “juiciness” and oral retention times of meat products [71].

In addition, κ-carrageenan as a linear sulphated polysaccharide is usually added to canned meats and pet foods as a gelatinizer. The thermal reversible gel properties of carrageenan affect the function of meat products, which are important to the sensory quality of meat. Carrageenan is dissolved in the whole meat during hot processing and solidified during cooling, which can improve water retention, texture, and consistency of comminuted meat products [73]. However, instead of chemically interacting with meat proteins, carrageenans increased the water-holding capacity of meat emulsions by physically holding water in the interstitial spaces of the protein–gel network. In practical application, other factors may affect the properties of meat protein as well. During the cooking process, changes in quaternary, tertiary, and secondary structure of proteins occur, which may further induce the changes in intra/intermolecular interactions. In sodium-reduced chicken breast myofibrillar protein gel, high pressure processing and calcium have been used to increase the water holding capacity [74]. Moreover, in emulsified meat products such as sausages, phosphate elimination affected the gelling characteristics of the products, and egg or pea addition remediated the quality loss [75]. More importantly, two transglutaminase enzymes were used in the restructured beef steaks to create covalent linking between meat proteins and improve the quality [76].

## 3. Proteins in Milk

### 3.1. Whey Protein

Whey proteins are milk proteins (mainly β-lactoglobulin and α-lactalbumin) that are widely used in food design due to a variety of functionalities, including: water binding, gelation, emulsification, and foaming [77]. In food processing, whey proteins are usually denatured due to heat treatments and thereby they aggregate, either in self-aggregation or with other food particles [78,79]. Through extensive research, it has been shown that the interactions between whey proteins and small molecules can change their molecular structures and the physico-chemical properties [79], supporting their applications in food.

**Figure 3:**
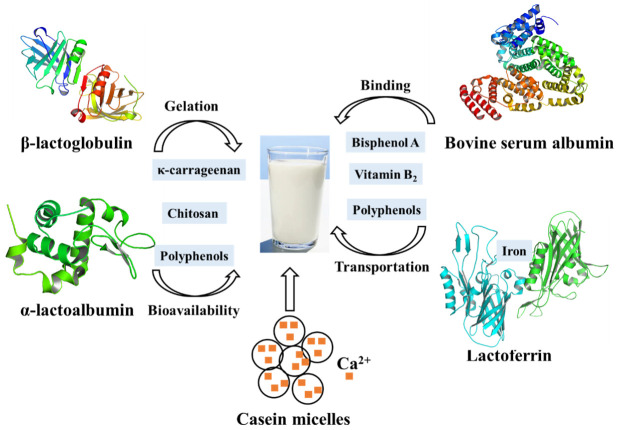
Main proteins in milk and their contributions to the quality of dairy products.

Most chitosans, which have been shown to effectively reduce the cholesterol blood level in animals and humans by strongly binding to the negatively charged bile acids, have the ability to interact with whey protein in milk [80]. At low pH, a weak carbohydrate-protein interaction is formed between chitosan and whey protein isolate, both of which are positively charged. When the pH is raised to 6.0, where the protein should be negatively charged, a much stronger interaction occurs due to electrostatic attraction between them, indicating that pH plays an overwhelming role in this complex system [10]. Therefore, these interactions can help separating whey protein during diary processing. Meanwhile, as a gelled agent, carrageenan is commonly used in the production of milk-based desserts. The addition of κ-carrageenan to whey protein-based desserts affects gel formation and caused an even higher storage modulus than milk-based desserts, possibly due to the interactions between whey proteins and κ-carrageenan at different pH values. At low pH, especially after heating, the whey protein will be exposed to a large number of hydrophobic groups to form large aggregates, which is the result of strong interactions with κ-carrageenan. In contrast, electrostatic repulsions between polysaccharide molecules and the negatively charged protein occurred at high pH. Therefore, optimal conditions help the production of highly digestible, light-colored whey protein components. In addition, phenolic-rich foods such as coffee and fruit juices are often consumed together with milk. Milk protein can affect the bioavailability of polyphenols through their interactions. Consistent with this idea, it has been shown that the phenolic acids (such as chlorogenic acid, ferulic acid, caffeic acid, and coumalic acid) can not only quench the fluorescence of β-lactoglobulin and α-lactalbumin by static quenching but also significantly affect the secondary structure of β-lactoglobulin and α-lactalbumin. More specifically, phenolic acids were proved to interact with the C=O and C-N groups of the structural subunits of whey protein [81]. As a consequence, the interaction between whey protein and other molecules not only affects the application of whey protein in the food industry but also the bioavailability (Figure 3).

### 3.2. Bovine Serum Albumin

Bovine serum albumin (BSA) is a large globular protein with two tryptophan residues, which is highly sensitive to the local environment [82]. Various studies over the past few decades have shown that in the presence of monovalent salts, BSA can form complexes with polysaccharides, surfactants, and other charged polymers [83]. Recently, interactions between BSA and small molecules have attracted considerable attention, due to their desirable and undesirable role in food stuff.

Bisphenol A (BPA), one of the most widely disseminated endocrine-disrupting chemicals and acrylamide AA, listed as a possible human carcinogen in carbohydrate-rich foods both have bad effects on wildlife and human health [84]. Research on the interaction between BSA and BPA or AA indicated that the binding of BPA and AA with BSA responded to the partition law and the secondary structure of BSA changed obviously when BPA and AA were present. The hydrophobic effect is the main factor that induces BPA binding to BSA, and the hydrogen bond between them promotes the interaction between AA and BSA [12]. Accordingly, the binding ratio of vitamin B2 to BSA has been reduced by more than 70% and almost one half, showing that BPA or AA inhibited the transport function of BSA. In addition, dietary flavonoids and stilbenes as important phytonutrient components found in vegetables, fruits, nuts, and tea have been widely studied. The flavonol moiety and the 5, 7-dihydroxylation at the A-ring are important structural features with significant antioxidant activity [85]. The binding ability of four differently substituted B-ring hydroxylation flavonols and a flavonol glycoside to BSA by quenching the intrinsic fluorescence of the protein was studied [86]. As expected, the binding affinity increased with the number of hydroxyl groups on the B-ring and the hydrogen bond force play an important part in the binding of flavonols to BSA. Moreover, polyphenols–protein interaction can be used to regulate the bioavailability of polyphenols. Smith et al. found that binding of polyphenols to albumin reduced their prooxidant activity [87]. Thus, interactions between them can weaken the antioxidant capacity of polyphenols, thereby affecting their application in food. Moreover, 5-Spiro-3′-piperidine-2″-spiro-3″-indole-4′,2″-diones (SPSD), an anti-tumor drug, has the ability to bind BSA through the hydrophobic force, van der Waals interactions, hydrogen bonds, and the electrostatic interaction, which can induce a conformational change of BSA. In addition, tricyclo [3.3.1.1(3, 7)] decane-1-amine (Amantadine) as an antivirotic drug can bind to BSA molecules and efficiently quench its intrinsic fluorescence [88]. In this way, the interaction between the Amantadine Schiff-Bases and BSA molecules can be strengthened by the methoxy group or the chloride atom. These works will contribute to the understanding of the mechanism of interactions between small molecules and BSA, and contribute to the future development of its potential biological, pharmaceutical, and physiological implications.

### 3.3. Casein

Caseins, the major proteins in milk, are a family of related phosphoproteins and consist of submicelles which in turn are aggregates of three calcium-insoluble proteins (αS1, αS2 and β-casein) and one calcium-soluble protein κ-casein [89]. The calcium-insoluble caseins are usually considered to be located predominantly within the micelles, while κ-casein is thought to coat the micelle, serving to stabilize the structure [90]. Additionally, the calcium-insoluble caseins are highly phosphorylated, and they interact with calcium phosphate interacts through their phosphate groups. In this way, the casein micelles carry a large number of (usually highly insoluble) calcium phosphate into milk and keep it suspended. However, the removal of subcritical amounts of Ca^2+^ from casein micelles with EDTA releases soluble casein, suggesting that Ca^2+^ removal initially dissociates weakly bound caseins from the micelle without destroying the micelle size determining framework. Generally, the minerals and caseins in milk are in dynamic equilibrium between the micellar (pelleted components) and serum phases (non-pelleted components) [91]. Temperature, pH, and mineral salts or calcium chelators can affect the partitioning of the components [91]. Studies of calcium-chelating agents showed that the amount of colloidal calcium phosphate removed from casein micelles limited the reversibility of calcium phosphate micelle dissolution and formation. Furthermore, addition of casein to milk has been proved to increase the Ca absorption in young rats [92].

**Figure 4:**
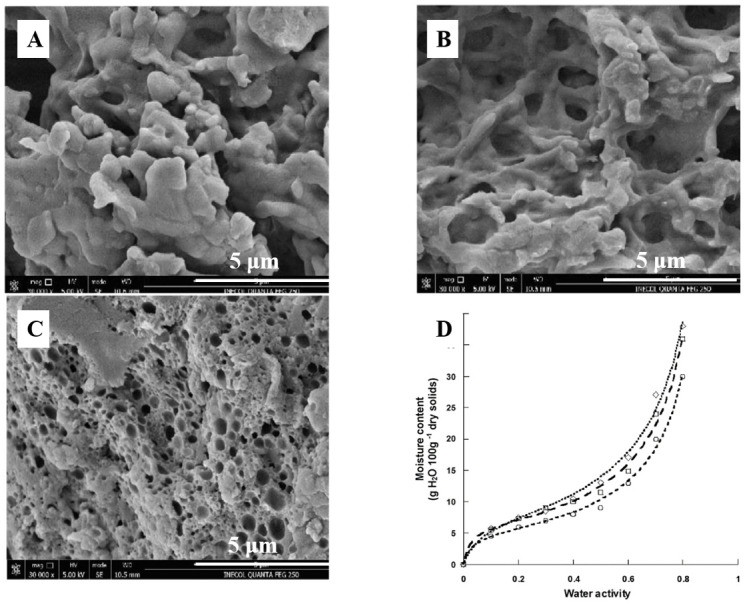
The ultrasound and freezing produced significant changes in the microstructure of casein, which produced a change in its physicochemical and functional properties. Micrographs obtained by scanning electron microscopy of (**A**) Unmodified acid casein (UAC), (**B**) Freezing with nitrogen (FMC), and (**C**) Ultrasound (UMC). Magnification is 30,000; scale bars are 5 μm. (**D**). Sorption isotherms of UAC (○), UMC (□), and FMC (◇). This figure was cited from reference [94].

On the other hand, casein has a wide range of uses in food industry, especially in cheese. The milk salts, Ca^2+^ and (PO_4_)^3−^, play an important role in the rennet coagulation of milk and in the structure and buffering of cheese by the neutralization of negatively charged residues on casein and aggregation of renneted micelles [93]. Addition of low concentrations of Ca^2+^ also increases gel firmness, which is important for the cheese texture. Moreover, the functional properties of casein can be changed by modification. Casein modified by freezing or ultrasound exhibited better emulsifying capacity and water absorption capacity, helping to extend the shelf life of the products (Figure 4) [94]. The partial replacement of casein in cheese by modified caseins can improve the microbial stability and shelf life of the cheeses. More interestingly, the interactions between casein and tannic acid have been further applied in food packaging, the results of which suggest that tannic acid is an effective crosslinking agent for casein protein, and can improve the water resistance of film [95].

### 3.4. Lactoferrin

Lactoferrin (LF), a glycoprotein that binds to Fe^3+^, was first found in milk and then in other human epithelial secretions and barrier body fluids. In breast milk, only 15% of LF is saturated by iron. Like the transferrin, lactoferrin has a molecular weight of 76 kDa, which binds two metal ions and is synergistic with two bicarbonate ions [11]. LF’s stronger iron-binding affinity makes it the only transferrin capable of retaining this metal over a wide range of pH values, even the extremely acidic pH [96]. The binding metals are not only the Fe^3+^ ions, but also Cu^2+^, Zn^2+^ and Mn^2+^ ions. LF has the ability to reversibly bind to Fe^3+^, so it has different three-dimensional conformations (Apo-LF or Holo-LF) according to whether it binds to Fe^3+^. Apo-LF can be rapidly degraded into small peptides by trypsin, while the iron-saturated form is relatively resistant to long-term trypsin digestion, producing only large fragments and retaining its iron-binding ability.

In addition, LF is a multifunctional protein because of its net positive charge and distribution in various tissues [29]. It is involved in a variety of physiological functions, including: regulation of iron absorption; antioxidant, anticarcinogenic and anti-inflammatory properties; immune response; and protection against microbial infection, which is the most widely studied function so far [29]. Due to the direct interaction with the bacterial surface, by carrying the Fe^3+^ ion, LF restricts the utilization of nutrients at the infection site by bacteria, and inhibits the growth of these microorganisms and the expression of virulence factors, thus achieving an antibacterial effect.

Overall, in terms of safety, bioavailability, and productivity, LF has been identified as a natural iron solubilizer and nutraceuticals for food products and plays an antibacterial role in the food industry.

## 4. Proteins in Egg

### 4.1. Lysozyme

As one of the least expensive forms of protein, fresh shell eggs are among the most nutritious daily foods. However, shell eggs’ internal quality deteriorates, and bacteria grow easily during storage. Egg-white lysozyme is an important component in the prevention of bacterial growth in hen eggs and is usually used as a preservative to inhibit the growth of deleterious organisms thus prolonging shelf life [97]. Lysozyme is a kind of enzyme that lyses the cell walls of certain Gram-positive bacteria as it splits the bond between N-acetylmuramic acid and N-acetylglucosamine of the peptidoglycan in the bacterial cell wall [98]. Despite the direct bacteriolytic action, it is reported that lysozyme had many other biological functions such as antiviral action to inactivate certain viruses by forming an insoluble complex, anti-inflammatory and antihistaminic actions, potentiation of antibiotic effects, and so on. Nevertheless, the peptidoglycan envelope of gram-negative bacteria is known to consist mainly of lipopolysaccharide (LPS) and is resistant to natural lysozyme, and lysozyme was not suitable for general antimicrobial use due to its limited lytic spectrum. However, it is not suitable for general antibacterial use due to its limited lysozyme spectrum. When lysozyme is used with other substances such as chitosan or glucan, its antibacterial spectrum may be enhanced [99]. Research on lysozyme-dextran conjugate prepared by Maillard reaction showed that it has obvious antimicrobial activity against both Gram-negative and Gram-positive bacteria. The antimicrobial activity of the lysozyme-dextran conjugate exhibits stable lysozyme antimicrobial activity for Gram-negative bacteria and excellent emulsifying properties to solubilize the outer membrane [100].

Furthermore, lysozyme–chitosan coating solutions could be effectively produced for use in preservation, extending the shelf life of eggs by delaying the loss of interior quality during storage [99]. Developing different perishable food coatings with different antioxidant and antimicrobial effects is the goal of future work.

### 4.2. Ovalbumin

**Figure 5:**
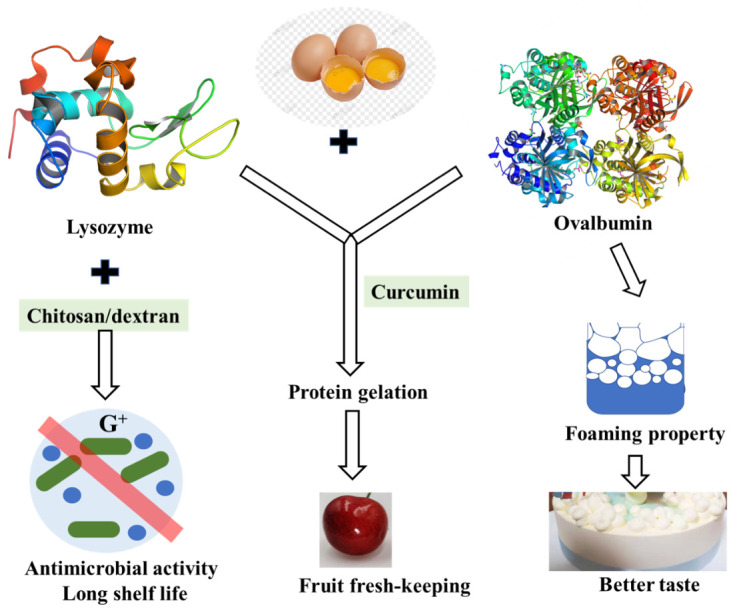
Functional proteins in eggs and their contribution to shelf-life and taste of food.

Ovalbumin is the main protein in egg white with good foaming, emulsifying, and gelation. It is found that the interaction between lysozyme and ovalbumin occurred in a chimeric mode, which may be caused by hydrophobic bonds [101]. The interaction between them is reversible, forming a homeostasis. Although this interaction reduced the bacteriolytic activity of lysozyme, the foaming properties and foam stability of egg white protein were decreased without lysozyme [102] (Figure 5). The relationship between egg white proteins remains to be further studied.

Egg albumin is rich in disulfide bonds and thiol groups, and thin films can be formed by alkali and heat treatment [103]. Compared with commercial polylactic acid (PLA) film, egg white protein (EWP) film, which is produced by an extrusion and rolling process, has higher water permeability, rigidity, heat resistance, and oxygen barrier, which can be used as part of semi-solid and solid food packaging materials [104]. Huang [105] mixed egg white protein and κ-carrageenan to prepare edible composite films, and found that, with the increase of egg white protein content, the disorder degree of the composite film increased, the elongation at break and light transmittance increased, but the oxygen permeability, water vapor permeability and water soluble decreased. Compared with the commercial film, EWP/κ-C composite film can effectively delay the oil rancidity. Tiimob [106] found that the addition of egg white protein into poly butylene adipate-co-terephthalate (PBAT)/polylactic acid (PLA) blends could make the resulting film have a certain antibacterial effect, showing the potential of egg white for manufacturing food packaging materials. It is noteworthy that, in the production of oat noodles, wheat noodles and pasta, the addition of egg white can lower the cooking loss and increase the springinesss and resilience by influencing the water absorption and texture [31].

## 5. Protein in Fishery Products

### 5.1. Collagen

Texture determines the acceptance of seafoods by consumers and thus determines the marketability of seafoods, so it is considered as one of the important quality attributes of seafood. As the main component of connective tissue membranes, collagen in fish product muscles is responsible for the integrity of the fillets. Unlike other muscle foods, however, fishery products usually remove stiffness more quickly, eventually leading to widespread extensive softening of the flesh [107]. The initial steps in deterioration of raw fish during its storage on ice consist of hydrolytic reactions catalyzed by endogenous enzymes, which produce nutrients that allow bacteria proliferation [108].

During refrigeration, proteolysis may affect the texture changes, so special attention should be paid to it. Although opinions differ on the effect of collagen on flesh softening, the destruction of fine collagenous fibrils in the skeletal muscle of cod may be caused by endogenous collagenases and other proteases [109]. In general, specific collagenases initiate the initial attack on collagenous tissue. Once the initial cleavage has been achieved, other nonspecific proteases can pursue an attack [110].

Additionally, attempts have been made to control the seafood texture by inactivating the related enzymes, for example, high hydrostatic pressure [107]. Moreover, collagen as a main part of fishery products has been extensively applied in the food system and cosmetics. More efforts should be made to improve the quality of fishery products by modifying collagens.

### 5.2. Polyphenol Oxidase

**Figure 6:**
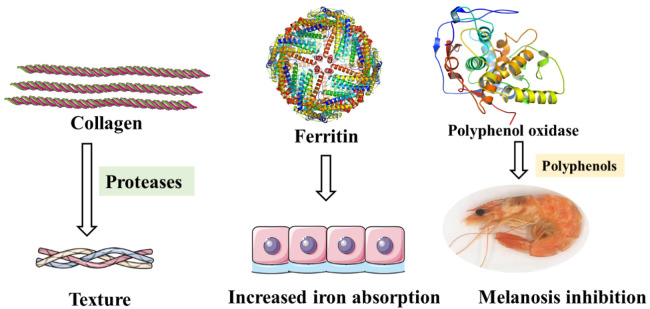
Specific proteins in fishery products that contributed to the texture, color, and nutrition.

Because of the existence of polyphenol oxidase, crustaceans such as shrimp, prawn, crab, and lobster are prone to melanosis. Polyphenol oxidizes phenols to quinones, followed by nonenzymatic polymerization and autooxidation of quinones, resulting in the production of high molecular weight dark pigments [38]. Although melanosis does not cause damage to the human body, it reduces the economic value of products to a certain extent. Sulfite-based compounds, their derivatives and polyphenols can inhibit melanosis. Among them, natural plant polyphenols are safer and have been widely studied [111]. Calvo [112] found that all polyphenol extracts showed higher polyphenol oxidase inhibitory activity than sulfite by comparing the inhibitory effect of polyphenol extracts from five halophytes on diphenol oxidase of white shrimp, and *Portulaca oleracea* extract was the most effective one (Figure 6).

### 5.3. Ferritin

Ferritin has a highly conserved conformation and is widely found in the animal and plant kingdom [113]. As an iron storage protein, ferritin plays a key role in maintaining cellular homeostasis. Moreover, in vertebrates, ferritin is responsible for resistance against oxidative stress, and increased level of ferritin is also a non-specific marker of inflammatory processes [114,115]. Similarly, in Pacific white shrimp, ferritin expression has been reported to be sensitive to pH treatment, which means ferritin expression levels may be indicators of the pH stress around the shrimp [116]. It is worth mentioning that purified recombinant ferritin cloned from black tiger shrimp *Penaeus mondon* can help to reduce the mortality in shrimp infected with *Vibrio harveyi* [117]. Exogenous injection of purified crustacean ferritin can help protect the hosts from microbial infection [118].

In New Caledonia, blue shrimp with orange gills has been observed in the past decade. The coloring appeared during processing and marketing, and significantly affected the quality and selling prices of shrimp. Lemonnier et al. showed that the coloration resulted from the iron that settled on the gill tissue surface [119], which suggested that free iron in the pond is bad for shrimp quality, and the intensity of the orange gills may be reduced by using ferritin to mobilize free iron. Additionally, ferritin from shrimp with high contents of iron inside can also be developed as iron supplements in the future.

### 5.4. Crustacyanin

**Figure 7:**
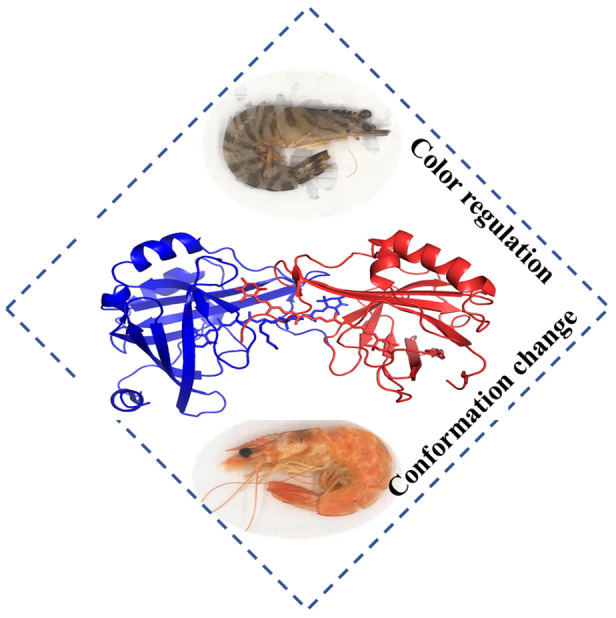
The interaction of crustacyanin and axtaxanthin affecting the color of shrimp.

In lobster crustacyanin, the bathochromic shift has attracted the attention of scientists for over 50 years. Color as a crucial factor affecting the quality of shrimp has been identified to relate with the function of crustacyanin (Figure 7). During cooking, the color of shrimp turns to red, mainly due to the denaturation of crustacyanin and release of astaxanthin. Therefore, the interactions between crustacyanin and chromophore seem to be important. In 2002, the first crystal structure of crustacyanin and astaxanthin were resolved, suggesting that the protein contacts and structural alterations may be responsible for color regulation [120]. In 2015, Gamiz-Hernandez et al. elucidated the mechanism underlying the color shift to be the electrostatic and steric effects of crustacyanin and astaxanthin [95]. Practical applications of this protein need to be further identified.

## 6. Conclusions

Proteins from animal food contain almost all kinds of necessary amino acids, which make them the good sources of protein. In natural food, the properties of protein are closely related to the texture, shelf-life, color, and taste of food. As the food industry develops, other molecules such as polysaccharides, polyphenols, and enzymes have been used to enhance the specific properties of protein, and thus improve the quality of food. Although proteins from meat, milk, eggs, and fishery products and the potential interactions between protein and other molecules affecting food quality have been summarized in this review, there are several aspects that need to be further investigated. Firstly, various kinds of molecules have been reported to improve the gelation property of meat; it is necessary to find the crucial molecules that affect quality. Secondly, although the proteins in milk have been extensively studied, there are still other enzymes or functional factors contributing to the quality of dairy products. Thirdly, enzymes responsible for autolysis are crucial for fishery products; thus, their identification and inhibition are significant. Therefore, in the future, many more efforts should focus on identification of proteins/enzymes, and the specific interactions between proteins/enzymes and functional factors based on their crystal structures.

## Figures and Tables

**Table 1:** Main proteins from animal sources and their relationship with product quality.

Food Sources	Main Proteins	Relationships with Product Quality	References
Meat	Myofibrillar protein	Form heat-induced gel Related to the water retention, chewiness and juiciness of meat products.	[13,14]
	Sarcoplasmic protein	Related to the color of meat products. Influence gel properties, such as water holding capacity. Indirectly affects meat tenderness.	[15,16]
	Connective tissue	Affect the texture, tenderness and water holding capacity of meat products.	[17]
	Hemoglobin	Soluble, foaming, emulsifying, colorant, and nutritional supplement.	[18,19]
	Plasma protein	Foaming, solubility, emulsifying, gelling properties, and fat binder.	[18,20]
Milk	Whey protein	Emulsification, foaming, gelling, water binding properties. Used as thickener, stabilizers, and fat substitutes in yogurt.	[21,22,23]
	Bovine serum albumin	Physiological model protein, foaming, emulsifying, gelling properties.	[24]
	Casein	Solubility, emulsifying, gelling, foaming, stability. Can be used to make food films.	[25,26,27]
	Lactoferrin	Biological activities: iron metabolism regulation, immune regulation, antibacterial, antitumor, anti-inflammatory. Can be used as iron solubilizer.	[28,29]
Egg	Egg white proteins	Gelling, water holding capacity, foaming and emulsifying properties. Can be used as thickener to give the product good taste and texture. Enhance the nutritional level. Can be used as food packaging film.	[30,31]
	Egg yolk proteins	Gelling, emulsification, film formation. Can be used as gelata, adhesive or color former to improve the nutritional value, flavor and taste of the product.	[32,33]
	Lysozyme	Antibacterial activity. Extend the shelf life of food.	[34]
Fishery products	Collagen	Used as emulsifier, foaming agent, stabilizer and nutrients in the product.	[35,36]
	Ferritin	Can be used as iron supplement.	[37]
	Polyphenol oxidase	Cause melanosis of crustaceans.	[38]
	Transglutaminase	Improve functional properties of proteins, such as gelation.	[39,40]
	Cathepsins	Promoting degradation of myofibrillar protein.	[41]

## Data Availability

Not applicable.
